# Pharmacists’ Perceptions on Safety Alerts of the Drug Utilization Review (DUR) in Electronic Health Records in a Tertiary Healthcare Hospital

**DOI:** 10.3390/pharmacy11040119

**Published:** 2023-07-20

**Authors:** Nouf Alshehri, Abdullah Alanazi

**Affiliations:** 1 Department of Health Informatics, College of Public Health and Health Informatics, King Saud Ibn Abdulaziz University for Health Sciences, Riyadh 11481, Saudi Arabia; 2 King Abdullah International Medical Research Center, Riyadh 14611, Saudi Arabia

**Keywords:** alarm fatigue, drug utilizing review, improving alarm, pharmacist, satisfaction

## Abstract

Electronic Drug Alarms and Drug Utilization Reviews (DURs) are crucial in improving patient safety by reducing the dispensing of contraindicated medications and minimizing adverse drug events. The DUR system often generates low-level alerts, making it challenging for pharmacists and doctors to discern more critical alerts. This can result in alert fatigue, causing burnout and jeopardizing patient safety. A cross-sectional study was conducted in a tertiary hospital to explore pharmacists’ perspectives and experience with the DUR system. This study aimed to identify their responses to alerts indicating a need to change the original prescription and the difficulties encountered. Out of all the participants, 85% had prior experience with DUR alerts. However, 40% of them expressed dissatisfaction with the alerts. Moreover, 88% of the participants received highly frequent DUR alerts, but only 40% believed that DUR alerts could identify rare adverse drug reactions. Additionally, only 27% of the participants altered their prescriptions based on alerts for the MAOI/serotonin modulator. The survey showed that 66% of participants believe improvements are necessary for the DUR system. Specifically, 77% of participants felt that more information is needed on overlapping prescriptions, 82% on patients with chronic diseases, and 82% on potential reactions caused by co-administration. At the same time, 75% raised concern about the need for backup for any server breakdown. Positive perceptions about DUR lead to changing the prescription in response to an alert. Therefore, improving the DUR system is crucial to prevent pharmacists from missing important alerts and to increase their awareness of clinically significant alarm signals. By doing so, we can optimize patient safety and contribute to providing high-quality healthcare services.

## 1. Introduction

As national demand for assessing medication outcomes and establishing drug-related clinical practices increased, the Medicaid Rebate Act of 1990 required Medicaid to introduce prospective and retrospective drug use evaluation (DUR) programs in 1993 [1]. In 2011, community pharmacies in England dispensed over 950 million prescription products, which continues to rise [2]. Due to the nature of the pharmacy setting, which can produce and provoke errors, there are many chances for drug errors [3].

The use of Electronic Health Records (EHR) systems has revolutionized healthcare and is a recommended solution to decrease medication errors, prevent potentially inappropriate prescribing, and ensure patient safety [4,5]. However, recent research and systematic reviews have yet to provide a conclusive verdict on the efficacy of these systems [6,7,8,9]. It has been observed that the existing pharmacy software may need to be improved and more regular attention should be given to safety warnings [10]. Additionally, there have been criticisms that these systems may increase the likelihood of errors [11]. Further investigation and improvements are necessary to ensure the safe and effective use of PMR systems in healthcare.

The drug utilization review system is a highly effective tool that significantly reduces medication-related errors and adverse events. This ultimately improves patient safety and the overall quality of care provided [1,12]. It is worth noting that while most Pharmacy Medication Review (PMR) systems are stand-alone, they can be networked together to support multiple terminals. These systems are designed to support individual pharmacy operations and utilize patient-specific graphics, medications, and clinical data to evaluate medication appropriateness. If enabled, they can also assist with inventory management and interface with other healthcare systems [10].

Pharmacists use PMR systems when inputting pharmacy orders to improve their clinical decision-making and ensure the safe dispensing of medications. The safety features of these systems notify users of any potentially harmful medications, drug combinations, interactions, clinical risks, errors, or adverse events [13,14,15]. However, there is a scope for improvement in the efficacy of these safety features [16,17,18,19]. Recent research shows that physicians prioritize simplicity and performance improvement while deploying Clinical Decision Support System (CDSS). Qualitative analysis also reveals four critical barriers to CDSS deployment: alert fatigue, inadequate accuracy, substandard user interface design, and insufficient customizability [20].

Certain countries, including the United States, face similar challenges with Drug Utilization Review (DUR) alert systems. It has been noted that notifications can be approved with minimal consideration by simply pressing a key, resulting in a heightened alert override [21,22]. This issue can prove problematic, as numerous DUR messages, even those of low significance, can contribute to “alert fatigue,” causing pharmacists and physicians to overlook or miss clinically crucial alerts [23]. There is currently inadequate data regarding how the DUR system affects pharmacists’ decision-making behavior and satisfaction and how to intervene to prevent medication errors during pharmacy order processing. Previous research has primarily examined the prescribing and administration phases [24,25,26].

It is essential to highlight that electronic drug alarms have proven to be instrumental in reducing adverse drug reactions, leading to lower mortality rates, fewer impairments, fewer hospitalizations, and decreased healthcare expenses. A 2013 study found that electronic medication record (EMR) system alerts can significantly decrease the distribution of contraindicated medications and adverse drug events caused by hyperkalemia and prescription errors [10]. Nevertheless, it is also critical to acknowledge that drug alerts may not always be beneficial, as low-value or false-positive alerts can potentially put patients at risk. Research shows that it took 331 notifications to prevent just one adverse medication occurrence. Overall, it is essential to balance the benefits and limitations of drug alerts and implement measures that ensure their effective utilization in clinical settings [22].

Discovering that physicians and prescribers disregard 90 percent of medication alerts and that more than half of the notifications are deemed irrelevant is disconcerting [27]. Although integrated decision support can prevent medication errors, not all mistakes are prevented due to alarms being disabled, infrequently reviewed, misconstrued, or improperly overridden. Pharmacists must exercise greater caution in ensuring medication safety to guarantee the accurate delivery of medicine and dosages to patients [28].

Extensive research has been conducted on the impact of overridden alerts on safety, yielding three notable publications. According to these studies, override rates of 57%, 90%, and 80% were observed, resulting in adverse outcomes in 2.3%, 2.5%, and 6% of cases, respectively [15,19,20]. Additionally, it was found that 0.8% of adverse outcomes could have been prevented by avoiding override in those cases [29]. Furthermore, filtering out unnecessary alert occurrences proves to be a more efficient use of time and resources. Over three months, 1,568 unnecessary alert occurrences were successfully averted, with only 106 “legitimate” alert instances filtered [30]. This approach helped to allow prescribers to avoid becoming overwhelmed by a barrage of warnings that were not clinically relevant. Ongoing research is focused on striking a balance between the number of alerts with their accuracy while also evaluating the effectiveness of the automatic filtering system [26,28].

According to a recent research study, it has been discovered that the acceptance rate of clinicians decreases significantly with each additional alert received during patient visits. The decline in acceptance rate is approximately 30% for each alert, and a mere 5% increase in the number of reiterated alerts leads to a 10% drop in acceptance rate [31]. The cognitive load on clinicians due to alert fatigue makes it difficult to locate relevant information among the less pertinent data [28,29]. A commonly encountered issue with alert systems is alert fatigue, which occurs when alerts are overly frequent, irrelevant, or repetitive, leading to a low signal-to-noise ratio. This phenomenon can result in mental exhaustion, causing critical signals to be missed along with clinically insignificant ones. While alert fatigue has not received extensive research attention, various factors, such as the appropriateness of treatment, clinicians’ confidence in their expertise and other information sources, inaccurate information, patients’ reluctance to alter their medication, and time constraints, may contribute to alert overrides. Furthermore, alerts that are too complex or require additional clarification regarding their clinical implications have also been observed to lead to overrides [30,31,32,33,34].

To fill this gap, the investigators conducted this questionnaire survey to determine the satisfaction and usefulness of drug utilization review features and alerts in ePMR systems at the moment of pharmacy order entry. Therefore, this study aims to identify pharmacists’ perspectives and experiences with the DUR system. It explores their responses to alerts indicating the need to change the original prescription and the difficulties encountered.

## 2. Materials and Methods

The investigators utilized a cross-sectional design questionnaire. Two pharmacists and three physicians with broad medication prescribing knowledge pretested and reviewed the original questionnaire. It asked respondents about their experiences with and recognition of the DUR alert system, their perspectives, satisfaction, and demographic information. The survey included 21 structured questions that took approximately 20 min to complete. Questions regarding recognition were rated on a 5-point Likert scale, while satisfaction was measured on a 100-point scale. This study’s populations are all pharmacists working under the pharmaceutical care department in both the in-patient and out-patient pharmacy sites who use the EHR system daily in a tertiary hospital. According to the Pharmaceuticals Care Services department, the estimated number of active daily pharmacist users is 284 pharmacists. The investigators used a confidence interval of 95% and a margin of error of 5%. Therefore, the minimum estimated sample size is 164 pharmacists. An e-mail survey was used to collect data for the DUR questionnaire from August to October 2021. At the start of the e-mail, a brief introduction was provided. “This study is to examine the pharmacist’s satisfaction with the drug utilization review using a questionnaire. This questionnaire includes 21 questions that will be distributed by the pharmaceutical’s Care department E-mail”. The survey was emailed twice in September and October 2021 to encourage more responses. Data collection concluded at the end of October.

## 3. Results

After thoroughly analyzing the survey responses, the key features were identified that qualified participants for recognition. Our study comprised a sample size of 106 participants, with a response rate of 64.6%. Our findings indicated that the responses were consistent across various practice areas and specialty classes, which suggests that our sample size was representative and lends further credibility to our results.

There were more female respondents (74.5%), and 65.8% of females were between 30–40 years of age, and 56% had a duration of service of 6–10 years. Furthermore, in-patient pharmacy sittings accounted for more respondents to the questionnaire, 51.9%, respectively; 30.3% had both in-patient and out-patient pharmacy experience. Of the 106 respondents, 84.9% (90) used the DUR system, while two did not, while 13.2% had no alert experience despite using the DUR system. Mean pharmacist satisfaction with the DUR system was 58.6% of 100 points, showing satisfaction overall. Moreover, 65% of the respondents believed that DUR alert frequency was high, whereas 11% answered that DUR system alerts have a low frequency.

Furthermore, 84.9% of the total respondents considered the DUR alerts helpful in preventing errors related to medication, whereas 11.32% did not consider them beneficial, with no significant difference between male and female respondents. Moreover, the majority of respondents, 93%, consider the DUR alert to have a high-frequency rate. Females and males (66% and 27%, respectively) reported a high frequency of DUR alerts, showing a significant difference (*p* = 0.0357). Frequent therapeutic duplication was the most frequent alert in 37.7% of responses. Respectively, 33% and 29% were because of frequent ingredient duplication and co-administration incompatibilities between prescriptions.

Additionally, 71% of pharmacists consider the type of alerts for co-administration, resulting in highly critical cases similar to those in light cases, while 87.7% of respondents agreed that important alerts were ignored because the number of alerts appeared too high. They showed no significant differences between males and females. Almost half of the respondents agreed that DUR alerts help recognize rare adverse drug reactions, 43% and 50% of pharmacists disagree, 36% female pharmacists agree, and 7% male agree—showing a significant difference between females and males (*p* = 0.0019). A total of 67% of respondents responded positively if alerts often suggest opposite clinical recommendations. While 57% of female pharmacists, agreed that the alert often gives recommendations opposite to clinical practice guidelines, 24% disagreed. On the other hand, 77% of male respondents agreed, and 5% disagreed with no significant difference. In addition, according to Table 1, 50.9% of the respondents found the DUR alert pop-ups challenging to comprehend, with 48.1% of females expressing the same sentiment.

To gather respondents’ thoughts on high-risk combinations, we inquired about alerts related to Monoamine Oxidase inhibitors (MAOI) and serotonin modulators. For instance, we mentioned alerts regarding the combination of selegiline/moclobemide and amitriptyline/nortriptyline. The result indicates that 48% of respondents said they would not change the prescriptions. In contrast, 27% of respondents will change their prescriptions based on the alerts. Thus, according to the data, more respondents preferred to refrain from modifying prescriptions with co-administration incompatibilities than dispensing them modified. There is a significant difference between individuals who would change medicine and those who would not (*p* = 0.0074).

Furthermore, 38.6% of respondents considered co-administration medication beneficial over adverse drug reactions and maintained prescriptions as a reason not to change the prescriptions. However, because of the pharmacist’s experience and knowledge that specific co-administration of medication does not cause significant adverse drug reactions, 27% will not change the prescription. Moreover, 17.9% will not change the prescription because patients take medications at intervals. Additionally, 16% of pharmacists will not change their prescriptions because patients have stopped taking previously prescribed medications (Table 2).

However, regarding responses to improvement in the DUR system, 88.6% of pharmacists agreed that the DUR system needs improvement by decreasing the frequency of alerts to reduce alert fatigue. Also, 94% of respondents believed that medical institutions need education on the DUR system. While 66% thought the DUR system needed to expand information to cover different specialties, 26% believed the opposite. Furthermore, 68.8% of the particpants responded negatively when asked if they were satisfied with the current system, while 25% were satisfied. However, more specific information on the substance of overlapping prescriptions was required by 77% of pharmacists. Table 3 describes the opinions of the participants on how to improve the DUR system.

Nevertheless, 58% of pharmacists believed there are diverse causes of overlapping prescriptions, including patients refusing to take their medication or elderly patients misplacing their prescriptions. Additionally, 75% believed in the importance of the existing backup system in server breakdown situations. Moreover, with a significant difference (*p* < 0.0001), 94% of responses were positive when asked if they needed more information about the level of alert risk. In addition, pharmacists need more information about potential reactions attributable to co-administration: 89.6% showed a significant difference from those who disagreed (*p* = 0.0027). Furthermore, pharmacists were queried about whether the DUR should furnish details on patients with chronic conditions who require ongoing medication. Out of the respondents, 82% agreed, indicating a notable contrast (*p* = 0.0647) with those who disagreed (refer to Table 4).

To determine what influenced pharmacists to change a prescription after receiving a DUR alert, logistic regression analyses were conducted. Results showed that female pharmacists were more inclined to change the prescribed drug (adjusted odds ratio (AOR), 0.008; 95% confidence interval (CI), <0.001–0.357) than male pharmacists. Moreover, the factors that contributed to changing the prescribed medication were pharmacists who agreed that DUR alerts helped prevent medication errors (adjusted odds ratio (AOR), 108.946; 95% confidence interval (CI), 1.653–>999.999). According to pharmacists, important alerts are often disregarded due to their excessive frequency; pharmacists agree that the DUR alert pop-ups can be difficult to comprehend and that ingredients are frequently duplicated (Table 4).

**Figure 1:**
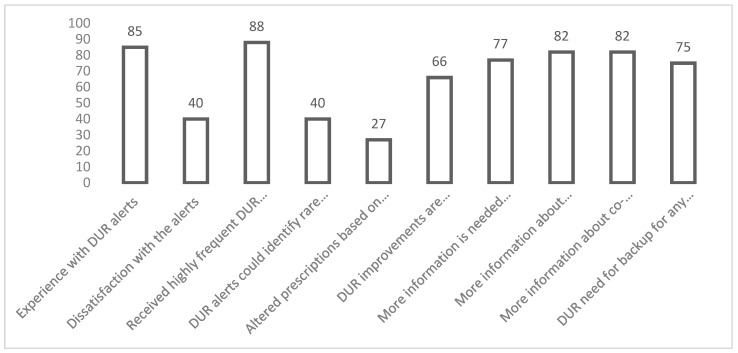
The main findings of the study.

Below is Figure 1, in which the study’s main findings are depicted.

## 4. Discussion

This study was undertaken to explore pharmacists’ perceptions of the drug utilization review (DUR) system, evaluate their perceptions regarding prescription modifications that are made following alerts, and identify the challenges they encounter at in- and out-patient pharmacies. The pharmacists’ perspectives are measured and presented through three themes: pharmacists’ satisfaction with DUR, the impact of DUR on Medication Safety, and DUR Usability.

### 4.1. Pharmacists’ Satisfaction with DUR

Most survey participants are satisfied with the DUR system, which received a rating of 71.5 out of 100 points in a nationwide survey to evaluate physician and pharmacist knowledge of DUR systems. This study found that pharmacists were dissatisfied with the generated alerts regarding the experienced volume and clinical practice. The DUR system reached a dissatisfaction rating of 60 out of 100 points. As an indication of alert acceptance and adherence to the DUR system, the prescription change rate can be viewed as a helpful sign [35,36]. According to data from physicians’ and pharmacists’ prescriptions from 2011 to 2012, it was reported that the rate of prescription changes due to incompatible drug co-administration alerts was 37.9% [37].

In this study, it was found that 27.36% of pharmacists are willing to change high-alert medication combination prescriptions based on DUR alert advice. Therefore, the need for a more intelligent and updated medication information system is increasing to increase the pharmacist’s acceptance and trust. From January to September 2006, Isaac et al. analyzed over 200,000 alert cases with the assistance of 2800 physicians; they discovered that notifications were generated in 6.6% of all electronic prescriptions, with physicians accepting 9.2 and 23% of drug–drug interaction (DDI) and allergy alarms, respectively [38]. In contrast, doctors who had previously accepted alerts were found to be less willing to take them once more. Valid alerts could be overlooked based on low vigilance or risk-level clinical judgments [28]. A total of 88% of pharmacists in this study approved that valuable alerts may be ignored because of high DUR alert frequency.

### 4.2. The Impact of DUR on Medication Safety

Despite low satisfaction, the data revealed that many out-patient practitioners utilizing electronic systems prevented severe adverse drug events by implementing alerts for drug allergies and drug–drug interaction alerts (DDI) by 47%. Furthermore, regardless of severity, 57% of healthcare practitioners believe that a monthly alert system helps to intercept prescription errors. A total of 22% believe drug warnings have stopped potentially serious errors or adverse effects. There are various recommended strategies to improve physician adherence to drug–drug interaction (DDI) alerts. Paterno et al. indicated that DDI alerts being differentiated from the CPOE based on severity level could help physicians’ sensitivity regarding alerts [39].

In 2004, Paterno et al. analyzed system alert data for two hospitals. They discovered that both hospitals utilized a service to verify drug–drug interactions (DDIs) in their computerized physician order entry system. DDI alerts were used to assess adherence across the two institutions. According to the study, hospital clinicians accepted the most severe warnings 100% of the time in hospitals with alert categorization. However, they only accepted 34% of the time without alert classification. The study suggested considering an alert-tiering technique in practice [39]. Therefore, more critical alerts must be distinguished from less important alerts to improve the existing system. These modifications are expected to raise the cancellation rate of prescriptions and rejection of medication preparations, following adding pop-up alerts to the system to inform pharmacists about the potentially harmful effects of certain prescription combinations. This improvement in pharmacist awareness of clinically relevant alerts will lead to better medication safety and patient care.

### 4.3. DUR Usability

To enhance the usability of the EHR system and increase pharmacist satisfaction with the DUR, a technique to accelerate the modification rate of prescription drugs should be proposed to produce relatively severe adverse drug alerts using a selection and engagement approach. During real-time evaluation, the DUR system must be updated to prevent pharmacists from neglecting unsafe or contradicting prescriptions resulting in significant medication errors and severe adverse drug events. Other research has created alert-tiering strategies to reduce alert fatigue, incompatible medicine co-administration, and conflicts related to drug age combinations. The survey’s participants also suggested areas for change that they believed should be emphasized.

Paterno et al. developed an alert-tiering system that sets a standard for drug pregnancy notification. These notifications are categorized and checked accordingly [39]. Therefore, adopting a new method for alert tiering is recommended, including (i) Tier 1 containing the most severe and dangerous events and should be presented as a hard stop, and (ii) Tiers 2 or 3 requiring an explanation from the physician or pharmacist, while (iii) the third tier offers only the required DDI risk information if requested.

Through surveys, pharmacists have provided valuable feedback regarding potential improvements to the DUR system. Among the suggestions put forth, some have proposed the removal of the duplication alert for chronic medication in cases where the prescribed duration has ended, and the physician must re-prescribe the same treatment. Conversely, others have suggested integrating a medication reference link within the alert, empowering pharmacists to make prescription changes based on clinical expertise.

It is important to acknowledge that there are certain limitations to the research conducted. One of the significant factors that contributed to this was the limited response from pharmacists, which could be attributed to their lack of interest in the DUR system. Additionally, there may be significant differences between the groups of pharmacists who respond to our survey and those who do not. When such a discrepancy arises, a nonresponse bias may exist. Moreover, it is important to note that the findings of this study may only apply to some hospitals since it was carried out in only one institution. Within the realm of research, a phenomenon known as acquiescence bias exists. This refers to the tendency of a respondent to agree with all statements or queries presented to them consistently. Furthermore, this is a one-center study, and generalizing the findings to other settings should be approached cautiously. Nevertheless, this study is descriptive, and data were collected through self-report responses. Therefore, it would be beneficial to conduct more rigorous studies on the impact of the DUR system and study how pharmacists respond to alerts through an observational approach. This will ensure that medication safety is improved and will foster greater acceptance of the healthcare system among providers, ultimately leading to enhanced patient care.

## 5. Conclusions

To maintain optimal standards of patient care, it is imperative to seamlessly integrate the drug review alert system into the pharmacists’ workflow. This entails addressing the issue of cognitive overload, implementing safety measures to enhance the system’s efficacy, and prioritizing efficiency and effectiveness. Further research is needed to determine the impact of the DUR system on pharmaceutical care in real-world settings using a thorough methodological approach. Clinical guidelines and best practices must be incorporated into an effective alert system. A comprehensive evaluation of the clinical feasibility of the alerts may be necessary, requiring a holistic approach. By focusing on these critical areas, all stakeholders stand to benefit from the successful implementation of this system.

## Figures and Tables

**Table 1:** Experience of DUR (Drug Utilization Review) alerts and satisfaction.

Variables	Female N (%)	Male N (%)	Total N (%)	*p* Value
Experience and satisfaction toward DUR alerts.				0.028
Any experience.	63 (79.75)	27 (100.00)	90 (84.91)	
Did not use the DUR system.	2 (2.53)	0 (0.00)	2 (1.89)	
Despite utilizing the DUR system, I have not experienced any experience with alerts.	14 (17.72)	0 (0.00)	14 (13.21)	
On a scale of 0–100, you are satisfied with DUR alerts by score.				0.024
Less than 60%.	45 (56.96)	19 (70.37)	64 (60.38)	
60% or more.	34 (43.04)	8 (29.62)	42 (39.62)	
Frequency of DUR alerts.				0.035
High.	66 (83.54)	27 (100.00)	93 (87.74)	
Low.	13 (16.46)	0 (0.00)	13 (12.26)	
DUR alerts can help identify rare adverse drug reactions.				0.002
Agree.	36 (45.57)	7 (25.93)	43 (40.57)	
Disagree.	30 (37.97)	20 (74.07)	50 (47.17)	
I do not know.	13 (16.46)	0 (0.00)	13 (12.26)	

**Table 2:** Opinions of respondents regarding alerts for monoamine oxidase inhibitor (MAOI)/serotonin modulator (high-risk drug interaction).

Variables	Female N (%)	Male N (%)	Total N (%)	*p* Value
Response.				0.0299
Not sure.	24 (30.38)	2 (7.41)	26 (24.53)	
Prescriptions are altered based on notifications.	18 (22.78)	11 (40.74)	29 (27.36)	
Alerts are not followed.	37 (46.84)	14 (51.85)	51 (48.11)	

**Table 3:** Opinions on improvements to the DUR system.

Variables	Female N (%)	Male N (%)	Total N (%)	*p* Value
Need to expand the coverage of the DUR system.				0.002
Agree.	61 (77.22)	9 (33.33)	70 (66.04)	
Disagree.	14 (17.72)	14 (51.85)	28 (26.42)	
I do not know.	4 (5.06)	4 (14.81)	8 (7.55)	
Further information regarding overlapping prescriptions is needed.				0.001
Agree.	65 (82.28)	17 (62.96)	82 (77.36)	
Disagree.	4 (5.06)	10 (37.04)	14 (13.21)	
I do not know.	10 (12.66)	0 (0.00)	10 (9.43)	
Back-ups for a breakdown of the server are needed.				0.001
Agree.	53 (67.09)	27 (100.00)	80 (75.47)	
Disagree.	8 (10.13)	0 (0)	8 (7.55)	
I do not know.	18 (22.78)	0 (0)	18 (16.98)	
Information about possible reactions that may arise from drug co-administration is needed.				0.032
Agree.	68 (86.08)	27(100)	87 (82.08)	
Disagree.	5 (6.33)	0 (0.00)	2 (1.89)	
I do not know.	6 (7.59)	0 (0.00)	17 (16.04)	
DUR should share information about patients who need ongoing medicine for chronic diseases.				0.0068
Agree.	60 (75.95)	27(100.00)	87 (82.08)	
Disagree.	2 (2.53)	0 (0.00)	2 (1.89)	
I do not know.	17 (21.52)	0 (0.00)	17 (16.04)	

**Table 4:** Factors that influence responses to DUR alerts.

Variable	Univariable Model			Multivariable Model		
	Unadjusted OR	95% CI	*p* Value	Adjusted OR	95% CI	*p* Value
Sex (Female).	0.429	0.169–1.088	0.07	0.008	<0.001–0.357	0.012
Age (>30).	3.186	1.002–10.126	0.049	19.881	0.009–>999.9	l0.44
Practice period as a pharmacist (>6 years).	5.062	1.106–23.178	0.037	230.52	0.007–>999.9	0.308
Any experience with DUR alerts and satisfaction.	1.489	0.384–5.776	0.565	2.147	0.014–340.07	0.767
More than 60 satisfaction rates of the DUR alerts.	0.482	0.190–1.221	0.124	1.025	0.153–6.86	0.979
Can DUR alerts prevent medication errors?	2.143	0.440–10.442	0.346	108.946	1.653–>999.9	0.028
The alerts for co-administrations showed both severe and mild symptoms.	0.784	0.179–3.443	0.747	38.425	0.315–>999.9	0.136
Many important alerts are being overlooked.	1.432	0.279–7.335	0.667	<0.001	<0.001–0.189	0.010
DUR alerts help find rare drug reactions.	1.226	0.484–3.107	0.668	>999.999	6.103–>999.9	0.013
The DUR alerts may contradict standard clinical guidelines.	0.523	0.192–1.420	0.203	1.195	0.035–41.100	0.922
The DUR alert pop-ups are difficult to understand.	0.590	0.243–1.438	0.246	>999.9	7.153–>999.9	0.008
Frequent ingredient duplication.	0.469	0.161–1.368	0.166	0.018	<0.001–0.81	0.038
Frequent therapeutic duplication.	0.460	0.163–1.295	0.141	1.460	0.054–39.64	0.822

## Data Availability

Not applicable.
